# Plasticity-related gene 3 promotes neurite shaft protrusion

**DOI:** 10.1186/1471-2202-14-36

**Published:** 2013-03-19

**Authors:** Tanja Velmans, Arne Battefeld, Beate Geist, Anna Soriguera Farrés, Ulf Strauss, Anja U Bräuer

**Affiliations:** 1 Netherlands Institute for Neuroscience, Meibergdreef 47, Amsterdam, 1105 BA, The Netherlands; 2 University of Basel, Department of Biomedicine, Mattenstrasse 28, Basel, CH 4058, Switzerland; 3 Institute of Cell Biology and Neurobiology, Center for Anatomy, Charité – Universitätsmedizin Berlin, Charitéplatz 1, Berlin, 10117, Germany

**Keywords:** Phospholipids, Cell differentiation, Cytoskeleton, Growth cone

## Abstract

**Background:**

Recently, we and others proposed plasticity-related gene 3 (PRG3) as a novel molecule in neuritogenesis based on PRG3 overexpression experiments in neuronal and non-neuronal cell lines. However, direct information on PRG3 effects in neuronal development and, in particular, its putative spatio-temporal distribution and conditions of action, is sparse.

**Results:**

We demonstrate here that PRG3 induces filopodia formation in HEK293 cells depending on its N-glycosylation status. The PRG3 protein was strongly expressed during mouse brain development *in vivo* from embryonic day 16 to postnatal day 5 (E16 – P5). From P5 on, expression declined. Furthermore, in early, not yet polarized hippocampal cultured neurons, PRG3 was expressed along the neurite shaft. Knock-down of PRG3 in these neurons led to a decreased number of neurites. This phenotype is rescued by expression of an shRNA-resistant PRG3 construct in PRG3 knock-down neurons. After polarization, endogenous PRG3 expression shifted mainly to axons, specifically to the plasma membrane along the neurite shaft. These PRG3 pattern changes appeared temporally and spatially related to ongoing synaptogenesis. Therefore we tested (i) whether dendritic PRG3 re-enhancement influences synaptic currents and (ii) whether synaptic inputs contribute to the PRG3 shift. Our results rendered both scenarios unlikely: (i) PRG3 over-expression had no influence on miniature excitatory postsynaptic currents (mEPSC) and (ii) blocking of incoming signals did not alter PRG3 distribution dynamics. In addition, PRG3 levels did not interfere with intrinsic neuronal properties.

**Conclusion:**

Taken together, our data indicate that endogenous PRG3 promotes neurite shaft protrusion and therefore contributes to regulating filopodia formation in immature neurons. PRG3 expression in more mature neurons, however, is predominantly localized in the axon. Changes in PRG3 levels did not influence intrinsic or synaptic neuronal properties.

## Background

Developing neurons extend long neurites to create a complex network. Key structures in the elongation of neurites are a highly motile growth cone and a stable neurite shaft, the latter generated by the suppression of protrusive activity. Neurite elongation and branching are governed by coordinated rearrangements of the cytoskeletal dynamics of actin and microtubules [1-3]. The actin cytoskeleton provides protrusive and contractile forces and microtubules form a polarized network allowing organelle and protein movement throughout the cell. Neurite protrusion requires a protrusive force to push the plasma membrane forward, as well as an addition of membrane materials. This includes microtubule fragmentation and local accumulation of F-actin for the formation of filopodia and lamellipodia and subsequent neurite branching, also called sprouting [4]. In addition, protein glycosylation has been proposed to play a crucial role in the regulation of neurite elongation and branching [5,6]: proteins that have been shown to regulate neurite outgrowth are regulated by N-linked oligosaccharides in folding and activity [7]. Many proteins involved in the coordination between microtubules and actin have been identified [8,9]. Among these, calpain, which is localized along the neurite shaft, prevents branching through the proteolysis of cortactin [10]. Cortactin, in turn, is an actin-binding protein that activates the actin-related protein 2/actin-related protein 3 complex (Arp2/3) to initiate filopodia formation. This signaling pathway is extensively studied and apparently not only involved in neurite extension [11]. The stabilisation or destabilisation of the cytoskeleton by intracellular signaling cascades is, in turn, controlled by extracellular cues. Among others, phospholipids such as lysophosphatidic acid (LPA) and sphingosin-1-phosphate (S1P) mediate cell motility, growth, proliferation and survival [12-15]. Modulation of phospholipid signaling occurs by members of the lipid phosphatase/phosphotransferase family (LPT, also known as PAP2) [16]. One LPT subgroup consists of the vertebrate-specific plasticity-related genes (PRGs, also termed lipid-phosphatase-related proteins, LPRs) [17]. One of the five known PRGs is PRG3 (LPR1), an integral membrane protein with six predicted transmembrane helices linked by intra- and extracellular loops [16,18]. Previous analyses showed that PRG3 mRNA is expressed as early as embryonic day 14 in cerebral cortex and hippocampus formation [18,19], suggesting a role in neuronal differentiation. Cortical PRG3 showed a gradient of expression with a higher level of expression in the dorsal cortex in comparison to the lateral cortex [19]. The mRNA level decreased to basal expression in adulthood [18]. Functional tests of PRG3 suggested a role during neurite outgrowth: PRG3 overexpression induced the formation of actin-rich, dynamic filopodia in several cell lines such as N1E-115, HeLa, and COS-7 cells [18,20]. Interestingly, this induction was not mediated by cdc42 or Rif, a Rho-family GTPase, and was independent of the Arp2/3 complex [20]. The neurite outgrowth was also not directly linked to phosphatase activity against any of the well-characterized LPT substrates, because no enzymatic activity for PRG3 was shown [18,20]. This study was designed to transfer findings on PRG3 actions to a neuronal, physiologically relevant developmental context and clarify the functional importance of endogenous PRG3. Our results indicate that i) PRG3 in immature neurons is located in all neurite shafts, where it determines neurite protrusions; ii) PRG3 expression in more mature neurons is predominantly axonal; iii) PRG3 expression patterns are not influenced by and do not influence synaptic currents; and iv) glycosylation of PRG3 is a prerequisite for membrane insertion.

## Methods

### Animals

Pregnant, postnatal and adult C57BL/6 mice obtained from our central animal facility were kept under standard laboratory conditions (12 hour light/dark cycle; 55% +/-15% humidity; 24°C +/-2°room temperature and water *ad libitum*, enriched and grouped) in accordance with German and European guidelines (2010/63/EU) for the use of laboratory animals. Approval of experiments was obtained from the local ethics body of Berlin (LAGeSO: T0108/11).

### Constructs

For overexpression of rPRG3 and mPRG3 (GenBank Accession no. AY299399, rat PRG3; NM_178756, mouse PRG3) in HEK293 cells, the following expression plasmids were used: pGFP-N1-rPRG3, pGFP-N1-mPRG3 and p3×FLAG-CMV-7.1-rPRG3. For Western blot analyses we used the following expression plasmids: mPRG1-eGFP, rPRG2-eGFP, mPRG4-eGFP, and rPRG5-eGFP. The sequence of the mature rPRG3 protein was subcloned from pGFP-N1-rPRG3 into p3×FLAG-CMV7.1 (Sigma-Aldrich, St. Louis, MO, USA) via Hind III and Sal I. The fragment was amplified using the following primers: 5´-AAG CTT ATG GCT GTA GAG AAC AAC-3´ and 5´-GTC GAC TCA GGT GAC TTC GGT CAT-3´ (Metabion, Martinsried, Germany). mPRG3 was amplified using the following primers: 5´-CTC GAG ATG GCT GTA GAA AAC AAC AC-3´ and 5´-GGT ACC CAG ACT TCG GTC ATG-3´. Ligation into pGFP-N1 (Clontech Laboratories Inc, Mountain View, CA, USA) was performed via XhoI and KpnI.

For knock-down of endogenous PRG3 (shPRG3) the following oligonucleotides were annealed and ligated into pSuper-eGFP (Oligoengine, Seattle, WA, USA): 5´-GAT CCC CCT TCA GAG GAA CCC AAG GCT TCA AGA GAG CCT TGG GTT CCT CTG AAG TTT TTG GAA A-3´ and 5´-AGC TTT TCC AAA AAC TTC AGA GGA ACC CAA GGC TCT CTT GAA GCC TTG GGT TCC TCT GAA GGG G-3´. As a control we used a shRNA target sequence against firefly luciferase. The following oligonucleotides were annealed and ligated into pSuper-GFP: 5´-GAT CCC CCG TAC GCG GAA TAC TTC GAT TCA AGA GAT CGA AGT ATT CCG CGT ACG TTT TTG GAA A-3´ and 5´-AGC TTT TCC AAA AAC GTA CGC GGA ATA CTT CGA TCT CTT GAA TCG AAG TAT TCC GCG TAC GGG G-3´. For the PRG3 shRNA rescue plasmid, four silent mutations were introduced into the region targeted by the shPRG3. Site-directed mutagenesis was carried out with QuikChange site-directed matagenesis kit (Stratagene, CA, USA). Forward primer 5´ –G GGA ATG TGT GTG GTT CAT AAC TTT AGG GGC ACA CAA GGC TCT CCT TCC AAA CC– 3´ and the reverse primer 5’ –GG TTT GGA AGG AGA GCC TTG TGT GCC CCT AAA GTT ATG AAC CAC ACA CAT TCC C- 3´ (mutated amino acids underlined) (Metabion, Martinsried, Germany) were chose. The mutated full-length mPRG3 gene was subcloned into the pDsRed2-N1 via XhoI and KpnI. Sequences of all the cDNAs were conformed by sequencing.

### Antibody generation and purification

**Figure 1:**
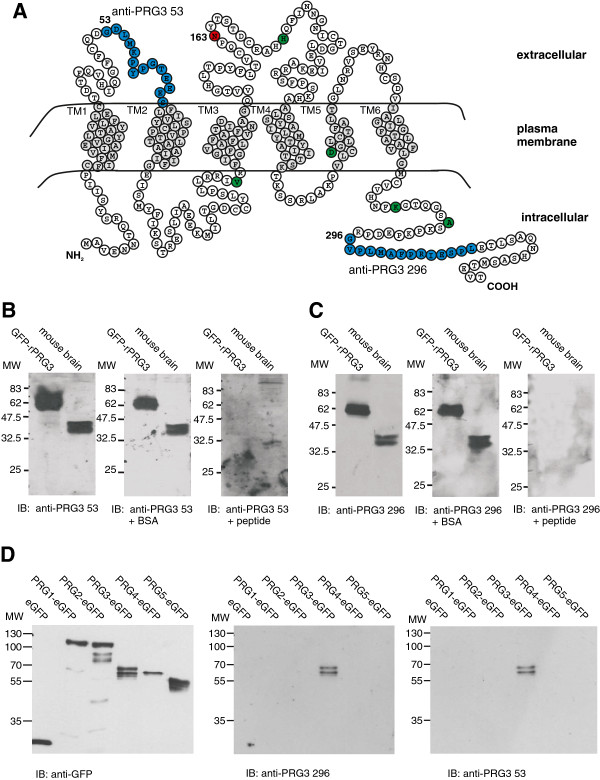
**Specific binding of PRG3 antibodies to intra- and extracellular domains.** (**A**) Schematic depiction of the amino acid sequence of rPRG3 with its predicted orientation in the plasma membrane. Transmembrane-spanning domains (grey) are labeled TM1-TM6 and the C- and N-terminal regions are located on the intracellular side. The putative N-glycosylation site at asparagine 163 is indicated in red, sites used to generate the PRG3 antibodies in blue, and loci that differ between PRG3 of mouse and rat in green. (**B** and **C**) Specificity analysis of the two anti-PRG3 antibodies revealed that anti-PRG3 53 (**B**) and anti-PRG3 296 (**C**) show similar immunoreactivity. HEK293 cell lysates overexpressing GFP-rPRG3 or endogenous PRG3 from mouse brain were probed with the antibodies under different conditions. Membranes probed with the native antibody (Western blot) showed a double band with either anti-PRG3 53 or anti-PRG3 296 (**B** and **C**, left). A two-hour preincubation of the antibody with BSA yielded the same result (**B** and **C**, middle), whereas a similar preincubation with the corresponding peptide abolished immunoreactivity (**B** and **C**, right). (**D**) Specificity analysis of the two anti-PRG3 antibodies revealed no cross reactivity to other PRG family members. Fusion proteins of several PRG family members with eGFP (PRG1, PRG2, PRG3, PRG4, and PRG5) were used in the Western blot analyses. Both used PRG3 antibodies (anti-PRG3 296 and 53) detected protein exclusively in the PRG3 lane as a double band.

Two polyclonal rabbit antibodies recognizing different sequences of PRG3 protein were generated. One was targeted to a sequence on the first extracellular loop (aa 53 – 66; anti-PRG3 53) and the other to a sequence on the intracellularly located C-terminus (aa 296 – 309; anti-PRG3 296). The sequences used to generate both antibodies were identical between rat and mouse PRG3 and are highlighted in blue in Figure 1A. The peptides (NH2-CGDLMKPYPGTEEES-COOH and NH2-CGVPLMAFPRIESPLE-COOH) for generating anti-PRG3 53 and 296 were synthesized by BioGenes (Berlin, Germany). The N-terminal cysteine residue was included for conjugation of the peptide to a carrier protein. Immunization and antibody production were performed by BioGenes. The antibodies were subsequently affinity-purified against the corresponding peptide with HiTrap NHS-activated HP columns (GE Healthcare, Buckinghamshire, UK) according to the manual. The antibody was dialyzed against PBS and stored at −20°C. The specificity of the antibodies was tested by immunoblot analysis. Here the antibody was preincubated with the corresponding peptide or BSA for control, for 2 hours at room temperature (RT) before adding the antibody to the membrane.

### Cell line transfection

HEK293 cells (human embryonic kidney) were routinely maintained at 37°C with 5% CO_2_ in DMEM (Invitrogen, Carlsbad, CA, USA) supplemented with 10% fetal calf serum (Pan Biotech, Aidenbach, Germany), 100 U/ml penicillin and 100 μg/ml streptomycin (Pan Biotech). HEK293 cells were plated in 6- or 12-well plates with a density of 20,000 cells/cm^2^ and transfected the next day with calcium phosphate precipitation: 4 μg DNA, 67.5 μl sterile water, 7.5 μl calcium chloride and 67.5 μl 2 × Hepes-buffered saline were mixed and either the whole amount added to one well for the 6-well plates or divided into two wells for the 12-well plates. One day after transfection the cells were used for further analyses.

### Primary mouse hippocampal neuron, astrocyte and microglial cultures

Hippocampal neurons were isolated from C57BL/6 mouse embryos at embryonic day 18. Hippocampi from several embryos were collected and washed twice in ice-cold HBSS (Invitrogen). The tissue was incubated in 4 ml HBSS and 400 μl trypsin (Invitrogen) for 15 minutes at 37°C, resuspended in plating medium (MEM supplemented with 10% horse serum, (both Invitrogen) 0.6% glucose, 100 U/ml penicillin and 100 μg/ml streptomycin). After dissociation, the neurons were plated in plating medium onto poly-L-lysine (Sigma-Aldrich) coated cover slips into 12-well plates or dishes with a cell density of 10,000 – 20,000 cells/cm^2^ or for a low-densitiy cell culture 5,000 – 10,000 cells/cm^2^. Three hours after plating, cells were washed twice with phosphate-buffered saline (1 × PBS) and incubated in Neurobasal A medium (Invitrogen) supplemented with 2% B27 (Invitrogen), 0.5 mM glutamine, 100 U/ml penicillin, 100 μg/ml streptomycin at 37°C and 5% CO_2_. For electrophysiological experiments neurons were plated with a density of 39,500 cells/cm^2^ and cultured in the absence of antibiotics. To block synaptic activity the medium was supplemented with 20 μM CNQX to block AMPA/kainate receptors, 100 μM DAPV to block NMDA receptors, and 4 μM gabazine to block GABA_A_ receptors. Medium was changed every second day. The treatment started from DIV 4 and lasted until DIV 14.

Astrocytes and microglia were prepared as previously described [21]. Briefly, mouse cortex (P1-3) was isolated and carefully homogenized. Digestion was performed by 10 minutes trypsin incubation. Dissociated glial cells were plated on poly-L-lysine-coated dishes and incubated at 37°C with 5% CO_2_. After two days the medium was refreshed and the cells were incubated until most of the microglial cells had detached from the astrocytes. The cells were shaken for two hours at 37°C and the remaining free-floating microglia were removed.

### Preparation of total protein lysates and recombinant proteins

HEK293 cells were washed with ice-cold PBS, lysed in a detergent-containing lysis buffer (50 mM Tris pH 7.4, 150 mM NaCl, 1% NP-40, 25 mM MgCl_2_, 10% Glycerol) containing protease inhibitors (Sigma-Aldrich), and sonicated 3 × 3 seconds. Subsequently, the lysates were centrifuged for 10 minutes at 14,000 × g and 4°C.

Mouse primary cells or organs were lysed in non-detergent-containing lysis buffer (20 mM Tris pH 7.5, 5 mM EDTA, 1 mM EGTA, 25 mM sucrose) with protease inhibitors. After sonication, the lysates from primary cells or organs were incubated on ice for 30 minutes and centrifuged for 5 minutes at 5000 × g and 4°C. Protein concentration of the supernatant was determined by a BCA Protein Assay Kit (Thermo Scientific, Rockford, IL, USA). To obtain recombinant protein, PRG1-eGFP, PRG2-eGFP, PRG3-eGFP and PRG5-eGFP were purified from transfected HEK-293 cells using the μMACS Epitope Tagged Protein Isolation Kit (Miltenyi Biotech, Bergisch Gladbach, Germany), as recommended by the manufacturer.

### Immunoblotting

Protein extracts from cells or tissues were subjected to 12% SDS-PAGE under reducing conditions and then blotted to a nitrocellulose membrane (Whatman, Dassel, Germany) by semi-dry protein transfer. Blots were blocked for 1 hour at RT in 10% non-fat dry milk diluted in PBS and then incubated overnight at 4°C with a 1:500 dilution of anti-PRG3 53 or 296, 1:1000 dilution of anti-FLAG (Sigma-Aldrich), 1:1000 dilution of anti-GFP (Clontech), 1:10000 dilution of anti-β-actin (Sigma-Aldrich), 1:5000 dilution of anti-GFAP (Millipore, Temecula , CA, USA) or 1:2500 dilution of anti-tuj1 (Covance, Berkeley, CA, USA). After three washes in 1 × PBS, the membranes were incubated with a 1:5000 dilution of an anti-mouse or anti-rabbit horseradish peroxidase-labeled antibody (GE Healthcare) for 2 hours at RT. For detection of Iba1 the membrane was incubated with a 1:2000 dilution of anti-Iba1 (Wako Pure Chemicals Industries, Osaka, Japan) in 5% non-fat dry milk, for 1 hour at RT. After three washes in PBS the membranes were incubated with horseradish peroxidase-labeled anti-rabbit secondary antibody 1:5000 diluted in 1 × PBS containing 5% non-fat dry milk for 45 minutes at RT. Immunoreactive bands were detected using ECL Western blotting detection reagents (GE Healthcare).The intensity of the Western blots bands was measured by using the MetaMorph Software, Version 6.2r6. The gray intensity of the control band was set to 100%. All other bands were calculated to the control. Beta-actin was used for normalization.

### Deglycosylation of PRG3

For detection of potential N-glycosylation, mouse brain or HEK293 total protein lysates were treated with peptide-N-glycosidase F (PNGase F, Roche Applied Science, Mannheim, Germany) as recommended by the manufacturer. Controls of mouse brain or HEK293 protein lysates were treated using the same process but without PNGase F.

### Point mutation of PRG3

Point mutation of the AS 163 of PRG3 from asparagine to glutamine within the expression plasmid p3×FLAG-CMV7.1-rPRG3 was performed with the QuikChange II Site-Directed Mutagenesis Kit (Stratagene, Cedar Creek, TX, USA) using the following primers: for 5´-CTGACA GTGTGCCAGCCACAATATACCAGTACAGACTGC-3´ and rev 5´-GCAGTCTGTACTGGTATATTGTGGCTGG CACACTGTCAG-3´ (Metabion) as recommended by the manufacturer.

### Electrophysiology

Hippocampal neurons were transfected at 11 or 12 days *in vitro* (DIV) using Effectene (Qiagen GmbH, Hilden, Germany) according to the manufacturer’s specifications. One to three days post transfection neurons were placed in a bathing solution consisting of (in mM): 124 NaCl, 4 KCl, 3 CaCl_2_, 2 MgCl_2_, 25 HEPES, 10 glucose; pH adjusted to 7.3 with NaOH. An inverted microscope (IM35, Zeiss AG, Oberkochen, Germany) equipped with phase contrast, a 25× objective and a 50 W mercury lamp was used to visualize neurons. Pipettes were filled with an intracellular solution comprising 120 K-gluconate, 10 KCl, 10 Na-phosphocreatine, 1 MgCl_2_, 1 CaCl_2_, 11 EGTA, 10 HEPES, 2 Mg^2+^ ATP and 0.3 GTP (in mM); pH set to 7.2 with KOH. Intracellular solution was stored on ice. Miniature excitatory postsynaptic currents (mEPSCs) were recorded at a holding potential of −70 mV and in a bath solution containing 0.5 μM TTX (Tocris, Bristol, UK) and 10 μM bicuculline (Tocris). Experiments were performed at RT (21 – 24°C) with an EPC-10 patch clamp amplifier (HEKA, Lambrecht, Germany) controlled by Pulse (v8.78, HEKA) software. All recordings were filtered with a 2.9 kHz Bessel-filter and sampled with a minimum frequency of 6.25 kHz. Analysis of intrinsic neuronal properties was performed using PulseFit (HEKA) and Origin 7 (Originlab Corp, Northampton, MA, USA). mEPSCs were analyzed with WinEDR and WinWCP (University of Strathclyde electrophysiology software; author John Dempster, UK). The automatic detection parameters were set to a threshold of 8 pA and a dead time of 15 ms. Although the threshold was about two times the noise level, false–positive events were detected and detection failures occurred, therefore a visual identification of events was performed. The amplitude of mEPSCs was calculated from the averaged baseline before the event to the 5 point averaged peak of the event. Data were compiled and presented using Origin. Statistical analysis was performed in Origin and StatView (Abacus Concepts Inc, CA, USA). ANOVA was used for multiple comparisons, student’s t-test for two normal distributed data sets and Mann–Whitney U test for two non-normal distributed data sets. Significance level was set to p < 0.05 (*). Data from individual experiments are presented as box plots with boxes showing the 25^th^ and 75^th^ percentile, as well as the maximum and minimum. A line crossing the box and individual data points indicates the mean.

### Immunostaining of cultured cell lines and primary neurons

Primary hippocampal mouse neurons and transfected neurons at 1, 4 and 14 DIV or transfected HEK293 cells were fixed in ice-cold 4% paraformaldehyde with 15% sucrose in PBS for 20 minutes at RT and washed three times with PBS. Permeabilization was performed by adding 0.1% Triton X-100, 0.1% sodium citrate in PBS for 3 minutes at 4°C. After washing the cells three times they were blocked with 10% fetal calf serum (FCS) for 1 hour at RT. Cells were then incubated with primary antibody in 10% FCS in PBS overnight at RT in the following dilutions: 1:200 anti-PRG3 296, 1:200 anti-tau1 (Chemicon, Temecula, CA, USA), 1:1500 anti-β-actin, 1:1500 anti-α-tubulin, 1:1000 anti-MAP2, anti-Tuj1 or 1:500 anti-FLAG (all Sigma-Aldrich), 1:5000 anti-GFP and anti-Na^+^/K^+^-ATPase (Abcam). After three washes with PBS, Alexa Fluor-conjugated secondary antibodies (Invitrogen), diluted 1:1500 in PBS containing 10% FCS, were added for 90 minutes at RT. Finally, cells were washed three times and mounted with Immu-mount (Thermo Scientific). Confocal images were acquired with an upright laser microscope (Leica DM 2500). Background correction and adjustment of brightness and contrast were performed using Leica confocal software. For co-localization studies z-stacks of both fluophore were sequentially acquired and average projected.

For immunofluorescence quantification the cellSens DimensionDesktop Version 1.4.1 XV 3.4 (Build 8624) software from Olympus was used. The green fluorescence (green for PRG3) intensity was measured in a region of interest (ROI, 4–5 μm^2^) as a maximum pixel fluorescence intensity drawn over three distinct MAP2 positive or tau1 positive areas for the PRG3 abundance on tau1 and MAP2 positive structures. All measurements were performed using three different immunostainings. Data analysis was performed using GraphPrism 4 (GraphPad Software Inc, La Jolla, CA, USA) from n = 105 ROIs for MAP2 and n = 111 ROIs for tau1 positive structures. Data are reported as mean +/− SEM.

The red fluorescence (red for endogenous PRG3) intensity was measured in a region of interest as a mean pixel fluorescence intensity drawn over five distinct areas, to quantify the knock-down (PRG3 shRNA versus control shRNA) efficiency in Tuj1 positive neurons. All measurements were performed using three different immunostainings. Data analysis was performed using GraphPrism 4 (GraphPad Software Inc, La Jolla, CA, USA) from n = 28 for PRG3 shRNA and n = 30 for control shRNA transfected neurons. Data are reported as mean +/− SEM.

### RNA extraction and cDNA synthesis

Organs were harvested from C57BL/6 mice and homogenized in TRIzol (Invitrogen) reagent. Primary neurons, microglial cells, or astrocytes were scraped in PBS, centrifuged for 5 minutes at 900 rpm and 4°C. Cell pellets were dissolved in TRIzol reagent. Total RNA was extracted using TRIzol reagent according to the manufacturer’s protocol. The concentration and purity of the isolated total RNA were determined by spectrophotometric analysis (Biomate 3 spectrometer, Fisher Scientific, Schwerte, Germany). cDNA was synthesized with 5 μg of total RNA using the High-Capacity cDNA Archive Kit (Applied Biosystems, Carlsbad, CA, USA) according to the manufacturer's protocol. As a control, reaction was performed without MultiScribe reverse transcriptase. cDNA was diluted 1:5 with RNase, DNase-free water and stored at −20°C. The quality of the amplified cDNA (with and without MultiScribe reverse transcriptase) was controlled by β-actin PCR. Identity and purity of primary cells was shown by quantitative real-time PCR with the neuron-specific class III β-tubulin (tuj1) as a marker for neurons, the glial fibrillary acidic protein (GFAP) as a marker for astrocytes and the ionized calcium-binding adaptor molecule 1 (Iba1) as a marker for microglial cells [22].

### Quantitative real-time PCR

Reverse transcriptase real-time quantitative polymerase chain reaction (qRT-PCR) was performed with the following gene expression assays: PRG3 (Mm00626670_m1), Iba1 (Mm00479862_g1), GFAP (Mm00546086_m1), tuj1 (Mm00727586_s1), GAPDH (Assay ID 4352932E) and β-actin (Assay ID 4352933E) (Applied Biosystems). For HPRT, separate primer and probe were used (Primer Mix: for 5´-ATCATTATGCCGAGGATTTGGAA-3´; rev 5´-TTGAGCACACAGAGGGCCA-3´ and probe 5´-TGGACAGGACTGAAAGACTTGCTCGAGATG-3´). The PCR was run on the ABI PRISM™ 7700 Sequence Detection System (Applied Biosystems) and the data obtained were processed by ABI PRISM software. Standard curves were produced with serial dilutions of cDNA from P10 mouse cortex with amplification efficiency between 90 and 100%. Data were normalized to two different house-keeping genes (β-actin and HPRT), which produced similar results. Each result is the average of three separate experiments and error bars represent SD.

### Morphological analysis

Primary neurons were transfected after 1 day *in vitro* (DIV 1) with the shPRG3 construct, the shPRG3 construct together with a PRG3 rescue mutant, or control shRNA targeting firefly luciferase using Effectene and analysed at DIV 4. For the morphological analysis fluorescence images were acquired with an Olympus BX50 microscope equipped with Metamorph software (version: 6.2r4, Molecular Devices Inc, Sunnyvale, CA, USA). Confocal images of primary neurons were acquired with an upright laser microscope (Leica DM 2500) equipped with a 63× objective (oil-immersion, 1.2 NA) using the 488 nm line of an argon-ion laser. Background correction and adjustment of brightness and contrast were performed using Leica confocal software (Leica Microsystems, Germany). The length of the longest neurite was measured in Metamorph. For subsequent analyses the numbers of branching points were determined by counting the number of neurites according to their respective order (ImgaeJ 1.43u, Java 1.6.0_10 32-bit). A neurite was classified as a “branch” when it was longer than 10 μm. The number of protrusions between 2 and 5 μm and between 5–10 μm were quantified as separate subgroups. For both groups protrusion length was measured from the tip to the intersection with the protrusion shaft. The mean total process length per neuron was evaluated by quantifying the length of all processes (ImageJ 1.45s, Java 1.6.0_35 64-bit). For averaging the protrusion length per neuron the major neurite was omitted. All measurements were performed using three to four different transfection preparations by investigators blind to the experimental conditions. Data analysis was performed using GraphPrism 4. Data are reported as mean +/− SEM, which are also presented as error bars in the figures. Significance was assessed using the two-tailed student's t-test for unpaired data at the given significance level (p) where: ** 0.001 < p < 0.01; * 0.01 < p < 0.05.

## Results

### PRG3 Requires N-glycosylation for insertion in the cell membrane and influencing of filopodia development

In order to identify the prerequisites for PRG3 activity, e.g. intracellular as opposed to plasma-membrane binding, we raised two polyclonal antibodies against PRG3. One was targeted at an extracellular loop (anti-PRG3 53) and the other at the intracellular C-terminus (anti-PRG3 296) (Figure 1A). Both new antibodies were shown to be sensitive as they detected the PRG3 protein i.) when endogenously present in mouse brain protein lysates, and ii.) when fused to GFP (GFP-rPRG3) and overexpressed in HEK293 cells (Figure 1B,C). Specifically, Western blot analysis showed one double band at 37–40 kDa in native brain lysates and another double band at 61–64 kDa in PRG3-overexpressing HEK293 cells (Figure 1B,C). The bands match the *in silico* predicted molecular weight of 36 kDa for PRG3 and 63 kDa for the GFP-tagged PRG3 (ExPASy, http://www.expasy.ch/tools). Both antibodies are specific, as PRG3 was undetectable after pre-incubation of the antibodies with their corresponding peptides (Figure 1B,C). Further, we detected no cross-reaction to other PRG family members with both antibodies (Figure 1D).

**Figure 2:**
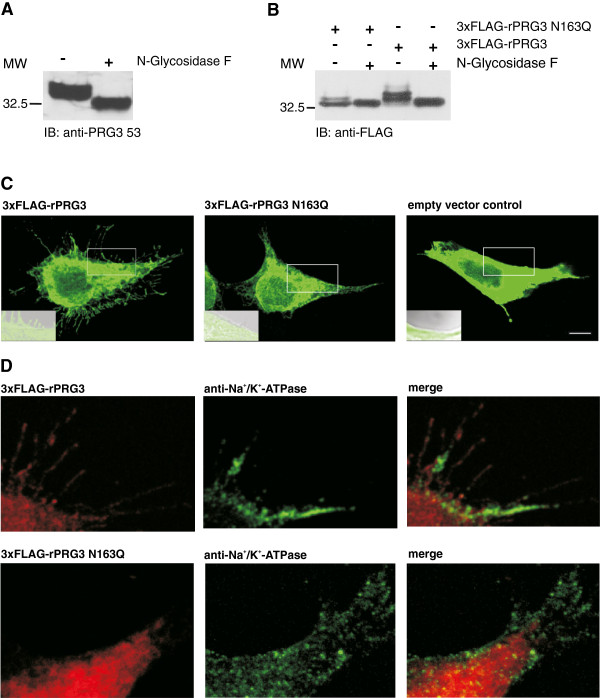
**N-glycosylation of PRG3 at asparagine 163 is required for membrane insertion and filopodia formation.** (**A**) Western blot of mouse brain lysates probed with anti-PRG 53 before and after incubation with N-glycosidase F. When treated with N-glycosidase F a band is detected at a lower molecular weight compared to untreated lysate, pointing to an N-glycosylation of PRG3. (**B**) Western blot of HEK293 cells after transfection with two different PRG3 constructs (3xFLAG-rPRG3 and 3xFLAG-rPRG3 N163Q). Native lysates (lanes 1 and 3) or lysates treated with N-glycosidase F (lanes 2 and 4) were probed with an anti-FLAG antibody. Mutation of asparagine 163 to glutamine (lanes 1 and 2) almost completely abolished N-glycosylation of rPRG3 whereas native protein was still N-glycosylated (lanes 3 and 4). (**C**) Confocal images of representative HEK293 cells transfected with either 3xFLAG-rPRG3 (left) or 3xFLAG-rPRG3 N163Q (middle) in comparison to control-transfected cell (empty vector) (right). HEK293 cells overexpressing the mutated rPRG3 construct show no increase of filopodia formation, which are prominently visible when HEK293 cells express the native rPRG3 variant. Cells were stained with a primary anti-FLAG and a secondary alexa488 antibody. Scale bar: 10 μm. White rectangles indicate the areas chosen for higher magnification displayed in the left lower left corner. Cells were visualized with fluorescence, laser transmission and combined in an overlay (shown). HEK293 cells expressing 3xFLAG-rPRG3 exhibited filopodia and rPRG3 was inserted in the membrane (left). In contrast, 3xFLAG-rPRG3 N163Q and empty vector control showed no localization in the plasma membrane and filopodia were not visible. (**D**) Co-localization images show wild-type PRG3 co-localized with the surface marker Na^+^/K^+^-ATPase, whereas mutated PRG3 shows no co-localization. Scale bar: 5 μm.

The existence of double bands in the immunoblots points to a putative glycosylation of the PRG3 protein. Indeed, hydrolyzation of N-linked glycan chains from mouse brain glycoprotein lysates with peptide-N-glycosidase F (PNGase F) [23] resulted in a single band with lower molecular weight (approximately 32 kDa) in the immunoblot using anti-PRG3 53 (Figure 2A). Furthermore, we identified a putative consensus N-glycosylation site at aa 163 within the second extracellular loop via *in silico* analysis of rat and mouse PRG3 (Figure 1A, marked in red; revealed by using NetNGlyc 1.0, http://www.cbs.dtu.dk/services/). Glycosylation is a posttranslational modification that plays a crucial role in the sorting and targeting of numerous proteins to their respective cell compartments [24]. We therefore hypothezised that altered PRG3 glycosylation could strongly influence its subcellular localization. To abolish N-glycosylation we point-mutated PRG3 (N163Q) by substituting asparagine for glutamine [25]. This N163Q mutation in its native, non-deglycosylated form showed only the lower molecular weight band of the endogenous protein together with a second band on the molecular weight of the deglycosylated form compared to wild-type 3xFLAG-PRG3 (Figure 2B). With PNGase F treatment the N163Q mutant showed a single band at approximately 32 kDa.

Next, we tested whether the N-glycosylation of PRG3 influences plasma membrane targeting by transfecting wild-type 3xFLAG-PRG3 and 3xFLAG-PRG3 N163Q into HEK293 cells. Overexpression of 3xFLAG-PRG3 induceed an increase in filopodia formation in comparison to control-transfected cells (empty vector), whereas the 3xFLAG-PRG3 N163Q mutant showed no alteration in the morphology compared to controls (Figure 2C). Additional evidence for membrane localization was obtained by staining with the surface marker Na^+^/K^+^-ATPase [26]. Mutated PRG3 failed to localize into the plasma membrane, in contrast to wild-type PRG3 that showed co-localization with the plasma membrane marker (Figure 2D).

Taken together, both endogenous PRG3 and 3xFLAG-PRG3 fusion proteins were glycosylated by N-glycosylation, which is required for the plasma membrane targeting of PRG3. PRG3 overexpression induces filopodia formations. However, the lack of PRG3 in the plasma membrane did not alter the cell morphology.

### PRG3 is mainly expressed in the brain and is developmentally regulated

**Figure 3:**
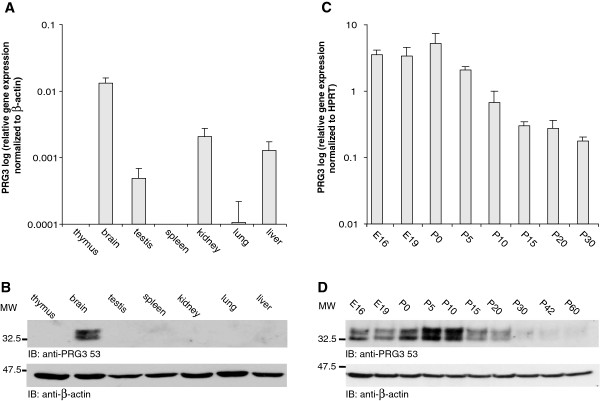
**PRG3 is mainly expressed in the brain and expression decreases during maturation.** (**A**) Quantitative real-time PCR of different mouse tissues (age P5) revealed the most prominent PRG3 mRNA expression in the brain. Data were normalized to β-actin. (**B**) Western blot analysis of protein lysates from various mouse tissues (age P5) shows a solitary signal in brain lysates (probed with anti-PRG3 53 antibody). No protein was detected in lysates from other tissues (top). As loading control β-actin was used (bottom). (**C**) Quantitative real-time PCR of mouse hippocampal tissue between E16 and P30 revealed age-dependent changes in expression levels. The highest PRG3 mRNA expression was detected around birth with a decline in expression levels until adulthood. Data were normalized to HPRT. (**D**) Western blot from total protein lysates of mouse hippocampal tissue between E16 and P60 (anti-PRG3 53 antibody). A signal was detected from E16 onwards with the strongest signal found around P5. From P10 to P20 a decrease in signal intensity appeared and after P20 the signal was hardly detectable (top). Anti-β-actin was used as loading control (bottom).

As the formation of processes is a prerequisite for the development of most tissues, we comparatively analyzed mouse PRG3 mRNA expression in different organs at P5 by quantitative RT-PCR (Figure 3A). This revealed the highest levels of PRG3 expression in brain and at least ten times lower levels in kidney, liver and testis. Additionally, lung tissue showed only a very weak signal that was just above the detection level. No PRG3 mRNA expression was detectable in thymus and spleen (Figure 3A). Interestingly, Western blot analysis only showed PRG3 protein expression in brain protein lysates (Figure 3B). There was no signal for any of the mouse tissues even after prolonged chemiluminescence exposure. The anti-PRG3 296 antibody yielded the same result (data not shown).

Given that PRG3 mRNA has been shown to be developmentally regulated in the hippocampal region [18], we evaluated the abundance of PRG3 mRNA and protein expression during development in this area. Between E16 and P5 we detected PRG3 mRNA at high levels in hippocampal tissue (Figure 3C). PRG3 mRNA expression then decreased more than 10-fold between P5 and P30 (Figure 3C). This expression pattern was reflected on the protein level: the level of PRG3 protein expression was high between E16 and P20, peaked between P5 and P10 and rapidly decreased until adulthood (Figure 3D). These data suggest that the PRG3 protein is exclusively expressed in brain tissue and that PRG3 might contribute to hippocampus development.

### PRG3 is prominently expressed in neurons

**Figure 4:**
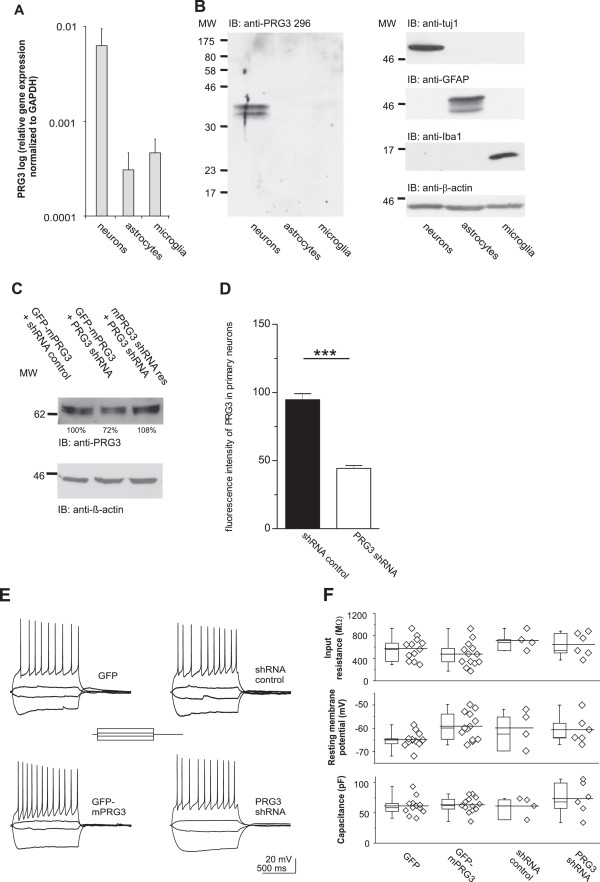
**PRG3 is neuron specific.** (**A**) qRT-PCR analysis revealed a ten times stronger PRG3 mRNA expression in neurons compared to astrocytes and microglia. Data were normalized to GAPDH. (**B**) WB from total protein lysates of primary brain cells. PRG3 protein was only detected in neurons. The membrane was reprobed with anti-tuj1, anti-GFAP, and anti-Iba1 to verify the quality of the lysates (right). Anti-β-actin was used as loading control. (**C**) Verification of the PRG3 shRNA and the shRNA resistant PRG3 construct by WB. GFP-mPRG3 was reduced when compared to control or to the PRG3 shRNA-resistant protein levels. Ratios below the WB bands refer to densitometric measurements of the gray intensity of these bands in relation to the control band. Anti-β-actin was used as loading control and for normalization. (**D**) Quantification of the efficiency of PRG3 shRNA. PRG3 shRNA (n = 28) expression reduced the endogenous PRG3 expression to more than 50% when compared to controls (n = 30). (**E**) Recordings of membrane voltage in response to a family of 1 second long square current pulses (see inset) from neurons expressing GFP (upper left), GFP-mPRG3 (lower left), control vector (upper right), or PRG3 shRNA (lower right). Irrespective of the PRG3 protein level, voltage responses including repetitive action potentials were roughly similar among all neurons. (**F**) Population data of intrinsic neuronal characteristics are displayed for the four groups of neurons depicted in D. Individual values for each neuron are shown at the right of each box plot. No differences in the resting membrane potential, input resistance, or capacity were found between GFP (n = 12), GFP-mPRG3 (n = 13), shRNA control (n = 4) or PRG3 shRNA-expressing cells (n = 6). An ANOVA resulted in p values of p = 0.07 (resting membrane potential), p = 0.15 (input resistance), and p = 0.51 (capacity).

To further specify the cellular abundance of PRG3 we used qRT-PCR to analyze its expression in primary cultured neurons, astrocytes and microglial cells. Primary cultured hippocampal neurons showed the highest levels of PRG3 mRNA, whereas the signal was over ten times weaker in astrocytes and microglial cells (Figure 4A). In Western blot analysis PRG3 expression was clearly detectable in primary neurons, whereas we found no PRG3 signal in cultured astrocytes and microglia (Figure 4B). The purity of primary cells was tested by immunoblotting with anti-tuj1, anti-GFAP and anti-Iba1 (Figure 4B). However, this neuron-specific expression pattern of PRG3 had no influence on intrinsic and general biophysical neuronal properties. We determined this by establishing different PRG3 levels in neurons via overexpression of PRG3 or shRNA-induced reduction of native PRG3 and compared the functional properties to the corresponding controls. To determine the PRG3 shRNA efficiency we analysed western blots after overexpression of PRG3 together with the shRNA in HEK293 cells. As specificity control of the PRG3 shRNA we generated a PRG3 shRNA-resistant construct, which was not effected by the PRG3 shRNA (Figure 4C). Furthermore, to demonstrate the neuronal specificity of the PRG3 shRNA we analysed and quantified the endogenous PRG3 level in primary neurons after transfection of the shRNA by immuncytochemistry (Figure 4D). The endogenous PRG3 level, detected by antibody staining, was reduced to less than 50% after transfection with the PRG3 shRNA. In both approaches we detected a clear reduction of PRG3 protein.

The neurons responded consistently to a family of hyper- and depolarizing currents (Figure 4E). In particular, suprathreshold currents evoked repetitive action potentials in all neurons. Subthreshold current injections were used to determine additional parameters of intrinsic neuronal excitability such as input resistance and resting membrane potential. Irrespective of the cellular amount of PRG3 these parameters appeared similar. Moreover, the whole cell capacity, an approximated measurement of the cell surface, did not differ between the experimental groups (Figure 4F). Hence, PRG3 is predominantly expressed in primary cultured hippocampal neurons, but does not profoundly interfere with the basal intrinsic functional properties of these neurons.

### Subcellular distribution of endogenous PRG3 in primary hippocampal neurons changes during development

**Figure 5:**
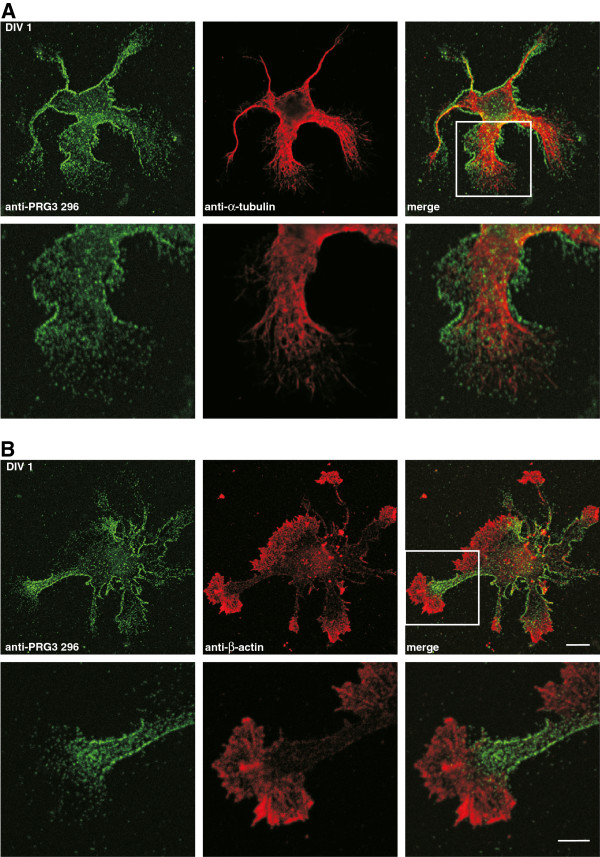
**Endogenously expressed PRG3 is found in the plasma membrane of primary hippocampal neurons after DIV 1.** (**A**) Confocal images of a representative primary hippocampal neuron fixed after DIV 1*.* Low (top row) and high (bottom row) magnification of this neuron showed that PRG3 (anti-PRG3 296, green) is not co-localized with α-tubulin (anti-α-tubulin, red) and is likely located in the plasma membrane. (**B**) Confocal images of a primary hippocampal neuron stained with anti-PRG3 296 (green) and anti-β-actin (red) after DIV 1. As in (**A**), PRG3 is located in the plasma membrane, but is only present in actin-rich regions and growth cones at low levels. Overview (top row), scale bar: 10 μm, higher magnification (bottom row), scale bar: 5 μm.

**Figure 6:**
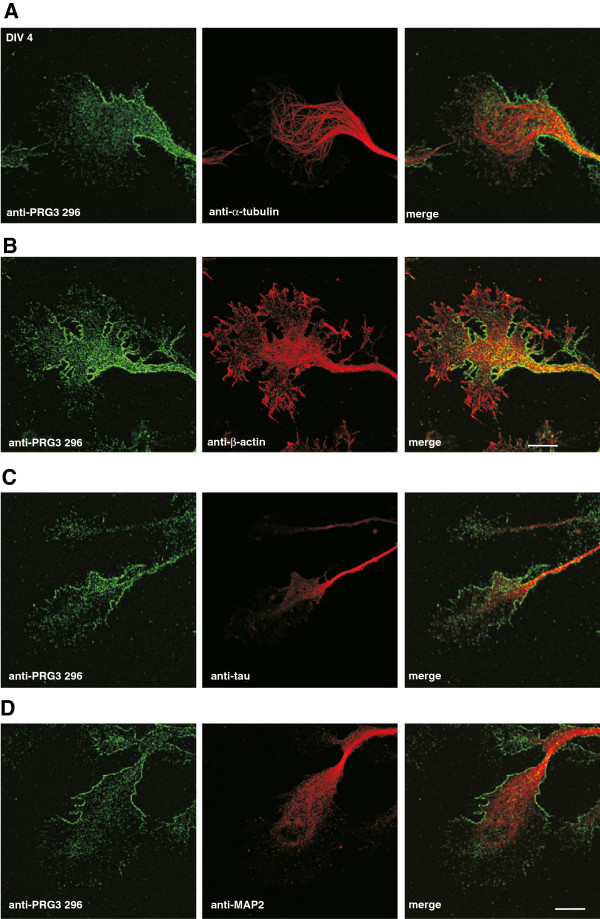
**Endogenous PRG3 is located in neurites and growth cones of primary hippocampal neurons after DIV 4.** (**A**) Confocal images of a typical extension of a hippocampal neuron at DIV 4, stained with anti-PRG3 296 (green, left) and anti-α-tubulin (red, middle). An overlay (right) shows that native PRG3 is found in all neurites, regardless of their fate. (**B**) Confocal images of a representative hippocampal neuron stained with anti-PRG3 296 (green) and anti-β-actin (red) showing that PRG3 is present in high amounts at the onset of the neurite, but only in a very small amount at the growth cone, which stained strongly for β-actin. (**C**) Confocal images of a hippocampal neuron stained with anti-PRG3 296 and anti-tau1 antibodies, and (**D**) a neuron stained with anti-PRG3 296 and anti-MAP2 show similar distribution of PRG3 at the neurite shaft, irrespective of the underlying structure. Scale bars: 10 μm.

Motivated by the prominent neuronal expression of endogenous PRG3 and its role in filopodia formation, we investigated the subcellular distribution of PRG3 in primary neurons at different developmental stages. After 1 day in culture, hippocampal neurons exhibited several undifferentiated, α-tubulin-containing processes of similar length (Figure 5A). During this initial phase of neuronal development, PRG3 appeared as a dotted, clear, strong signal in the plasma membrane and as weaker intracellular signal (Figure 5A and 5B). Beta-actin was strongly localized in growth cones and lamellipodia. Interestingly, PRG3 was expressed all over the plasma membrane except in the growth cones and lamellipodia, where ß-actin was located (Figure 5B). The distribution of PRG3 was uniform in more mature polarized neurons (DIV 4, Figure 6). Here, the microtubule cytoskeleton turns into parallel oriented bundles to form a nascent neurite shaft (Figure 6A) tipped by an actin-rich growth cone (Figure 6B). PRG3 was expressed in the plasma membrane along the neurite shafts, whereas β-actin-rich areas of the growth cone lacked PRG3 expression (Figure 6B). At this developmental stage, the neurons had established a single axon and several dendrites. PRG3 was present in the neurite shafts of axons, as shown by co-localization with tau1, an axonal marker protein, (Figure 6C) as well as in dendrites, as shown by co-localization with MAP2, a dendritic marker protein (Figure 6D).

**Figure 7:**
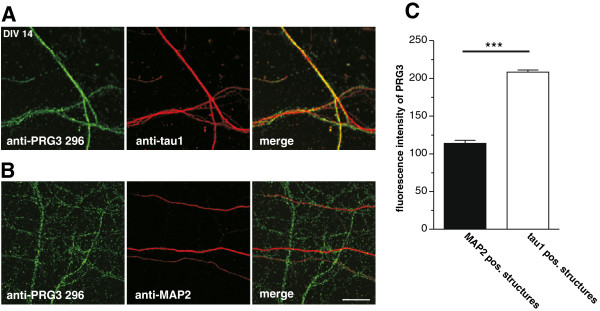
**After DIV 14 PRG3 is mainly found in axons of primary hippocampal neurons.** (**A**) Confocal images of axonal structures from hippocampal neurons stained with anti-PRG3 296 (green) and anti-tau1 (red) reveal that PRG3 is co-localized with these structures. (**B**) Confocal images of dendritic extensions stained with anti-PRG3 296 (green) and anti-MAP2 (red) show that PRG3 is not localized in dendrites. Scale bar: 10 μm, also applies to A. (**C**) Analyses of the PRG3 abundance on tau1 and MAP2 positive structures measured as the fluorescence intensity signal. The fluorescence intensity of PRG3 in tau1 positive structures (n = 111) is significantly higher compared to the PRG3 fluorescence intensity in MAP2 positive structures (n = 105) (p < 0.0001).

However, in neurons at DIV 14, when an extensive network of synaptic connections is established, PRG3 was predominantly expressed in axons (Figure 7A) as shown by co-localization with tau1. In contrast, neurites that were positive for MAP2 (Figure 7B) showed hardly any PRG3 expression at this time point. To measure the abundance of PRG3 in axons and dendrites, the fluorescence intensity of PRG3 on tau1 or MAP2 positive structures were analyzed. The PRG3 expression on MAP2 positive structures is only half the amount as on tau1 positive structures (Figure 7C). In summary, PRG3 was present in all neurites after the beginning of axogenesis at DIV 4, but shifted towards predominant axonal expression by DIV 14.

### PRG3 Knock-down in young primary hippocampal neurons decreases the number of neurites

**Figure 8:**
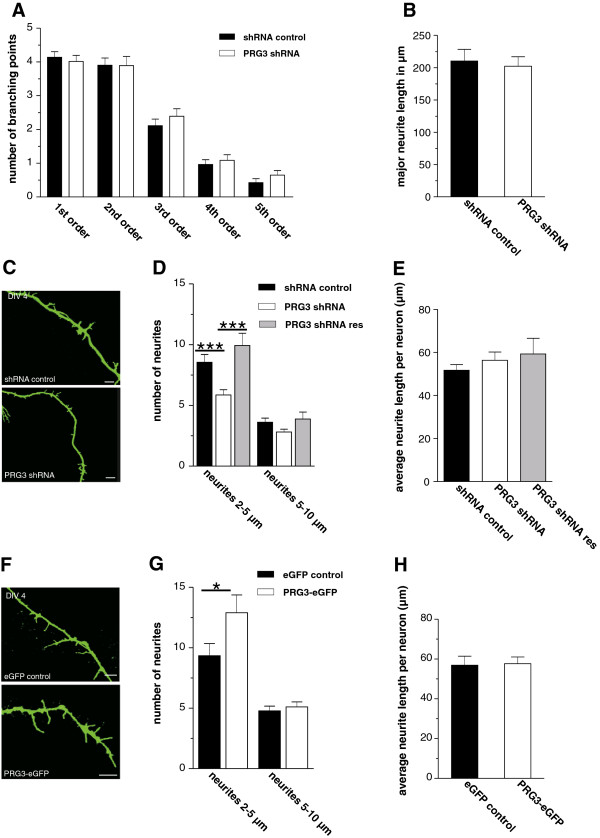
**PRG3 knock-down reduces the formation of small neurites.** (**A**) Primary neurons were transfected with PRG3 shRNA or control shRNA at DIV 1 and morphologically analyzed at DIV 4. Determination of branching points according to branching order revealed no differences between the two groups (n = 80). (**B**) Axon length measurement, in which the longest neurite of the transfected neuron was measured (n = 32). (**C**) Confocal images of representative transfected primary neurons showed a decreased quantity of small neurites in PRG3 shRNA-expressing neurons compared to controls. Scale bar: 5 μm. (**D**) All neurites within one neuron were grouped between lengths of 2–5 μm and 5–10 μm and are presented as subgroups. PRG3 knock-down decreased the number of neurites between 2–5 μm (p = 0.0006) but not in the number of neurites with a length between 5–10 μm. We co-transfected primary neurons with a PRG3 rescue plasmid together with the PRG3 shRNA. The double transfected neurons show neither a reduction of neurites with a length between 2–5 μm nor neurites with a length between 5–10 μm. (**E**) PRG3 knock-down did not affect the average neurite length per neuron as revealed by comparing PRG3 shRNA expression neurons to controls or to the PRG3 shRNA resistant construct. (**F**) Confocal images of representative transfected primary neurons showed an increased quantity of small neurites in PRG3-eGFP-expressing neurons compared to controls. Scale bar: 5 μm. (**G**) PRG3 overexpression leads to an increase in the number of neuritis with a length between 2–5 μm (p <= 0.0489). No effect was detectable in the number of neuritis with a length between 5–10 μm. (**H**) The average neurite length for each neuron was unchanged for PRG3-eGFP overexpressing cells and respective controls.

Because filopodia formation is a crucial step in neurite outgrowth, PRG3 might influence cell morphology. To investigate the effect of endogenous PRG3, primary neurons were transfected with PRG3 shRNA and morphologically analyzed. We neither observed a difference in the number of branching points (Figure 8A, Table 1) nor in the length of the longest neurite (Figure 8B, Table 1). However, shRNA-induced knock-down of PRG3 expression decreased the number of neurites with a length between 2 and 5 μm (t-test, p = 0.0018, Figure 8C,D, Table 1), whereas neurites with a length between 5–10 μm were not changed in number. Additionally, the average neurite length per neuron showed no alteration between PRG3 knock-down and control (Figure 8E, Table 1). To confirm that the observed neuronal phenotype was a specific result of the PRG3 shRNA expression, the PRG3 shRNA knock-down neurons were co-transfected with an shRNA-insensitive PRG3 construct. The numbers of neurites with a length of 2–5 μm in neurons co-expressing PRG3 shRNA and shRNA-resistant PRG3 was not different from that in those expressing control shRNA. Thus, overexpression of shRNA-resistant PRG3 in PRG3 knock-down neurons completely rescued the reduction of neurites with a length of 2-5μm (Figure 8D,E, Table 1).

**Table 1:** Data of Figures 8A, B, D, E, G, and H

**8A**	**shRNA control (n=80)**		**PRG3 shRNA (n=82)**			
	Mean	SEM	Mean	SEM		
1st order	4.138	0.1666	4.012	0.1799		
2nd order	3.900	0.2154	3.890	0.2733		
3rd order	2.113	0.1949	2.390	0.2214		
4th order	0.9625	0.1394	1.085	0.1648		
5th order	0.4250	0.1163	0.6463	0.1359		
**8B**	**shRNA control (n=32)**		**PRG3 shRNA (n=32)**			
	Mean (μm)	SEM (μm)	Mean (μm)	SEM (μm)		
Major neurite	210.50	17.86	202.20	14.75		
**8D**	**shRNA control**		**PRG3 shRNA**		**PRG3 rescue mutant**	
	Mean	SEM	Mean	SEM	Mean	SEM
Neurites 2–5 μm	8.551 (n=89)	0.645	5.862 (n=94)	0.426	9.938 (n=16)	0.9978
Neurites 5-10μm	3.612 (n=67)	0.338	2.806 (n=72)	0.2387	3.875 (n=16)	0.5764
**8E**	**shRNA control**		**PRG3 shRNA**		**PRG3 rescue mutant**	
	Mean	SEM	Mean	SEM	Mean	SEM
neurite length	51.71 (n=60)	2.647	56.29 (n=61)	3.845	59.28 (n=16)	7.253
**8G**	**eGFP control**		**PRG3-eGFP**			
	Mean	SEM	Mean		SEM	
Neurites 2–5 μm	9.345 (n=58)	01.005	12.90 (n=58)		1.474	
Neurites 5-10μm	4.776 (n=58)	0.3997	5.103 (n=58)		0.4179	
**8H**	**eGFP control**		**PRG3-eGFP**			
	Mean	SEM	Mean		SEM	
neurite length	56.88 (n=60)	4.497	57.59 (n=60)		3.389	

We also performed opposite analyses by overexpressing PRG3 in young (DIV 4) primary neurons. Here, we demonstrated an increase in the number of neurites with a length between 2–5 μm, but no change in number of neurites with the length of 5–10 μm (t-test, p = 0.0489, Figure 8F, G, Table 1). However, the average neurite length per neuron was not affected by PRG3 overexpression (Figure 8H, Table 1). This set of data suggests that PRG3 is involved in the induction of protrusions at the neurite shaft.

### PRG3 Distribution is not influenced by and does not affect synaptic currents in cultured Hippocampal neurons

**Figure 9:**
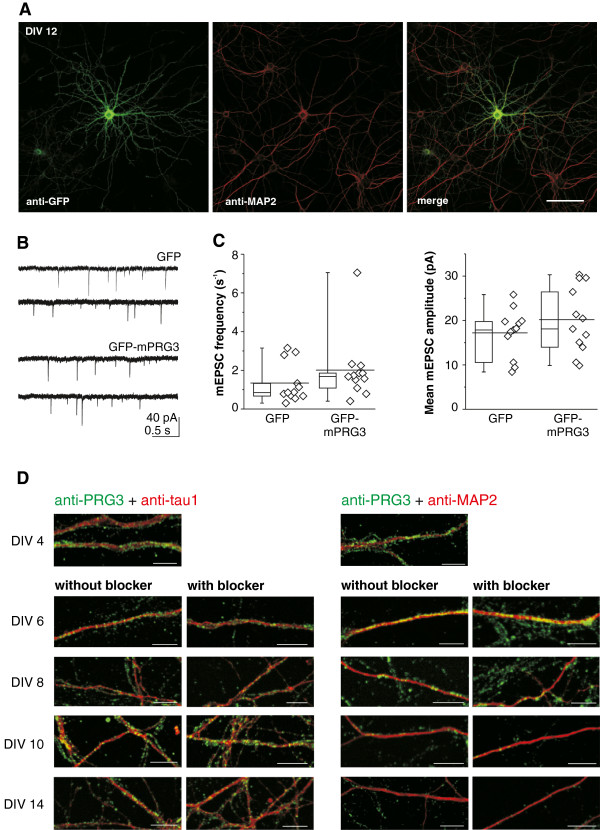
**Synaptic activity is neither altered by different dendritic PRG3 levels nor changes in PRG3 distribution.** (**A**) Image of a representative hippocampal neuron shows an evenly distributed PRG3 signal (anti-GFP) when overexpressed. Scale bar: 75 μm. (**B**) Representative current recordings from cultured hippocampal neurons either overexpressing GFP (native PRG3 level; control) or overexpressing a GFP-mPRG3 construct. mEPSCs were pharmacologically isolated (see Methods) and recorded at −70 mV. (**C**) Box plots of mEPSC frequency and mean amplitude box plots, as well as distribution of individual values. (Left) mEPSC frequency measured for each neuron is summarized and displayed as a statistical plot. Individual values are shown to the right of each box. No difference was observed (Mann–Whitney U test, p = 0.16, n = 12). (Right) For each neuron all mEPSC amplitudes were averaged. The mean amplitudes for each neuron are shown for either GFP (control) or GFP-mPRG3-overexpressing neurons. No difference was observed, regardless of the PRG3 level (unpaired t-test, p = 0.27, n = 12). (**D**) Immunocytochemistry analyses of PRG3 expression distribution on tau1 and MAP-2 positive structure with and without activity blockers. This treatment from DIV 4 to DIV 14 in culture did not change in the shift of PRG3 from dendrites to axons. Scale bar: 5 μm.

The PRG3 shift occurred in time and space in parallel to the beginning synaptogenesis of hippocampal neurons in culture [27]. We thus hypothesized first that the dendritic attenuation of PRG3 might ease stabilize synapse formation or second that developing synaptic inputs contribute to the PRG3 shift. An overexpression system was particularly suitable for investigating the first hypothesis because differences in expression levels between axon and dendritic structures seen in endogenous PRG3 protein distribution (Figure 7A,B) were not detectable (Figure 9A). We selected DIV10 as time point of transfection, because the PRG3 shift reached a steady state and synaptogenesis was still on a low level before the steep increase from DIV 14 on [27]. At DIV 12 we somatically recorded miniature excitatory synaptic currents (mEPSCs) of PRG3-overexpressing neurons (exemplified in Figure 9B) and respective controls. The mEPSCs were pharmacologically and electrically isolated. This was achieved in the presence of 0.5 μM tetrodotoxin, 10 μM bicuculline and a holding potential of −70 mV (Figure 9B). In the case a dendritic persistence of PRG3 interfered with proper synapse formation, we expected differences in incoming signals. However, neither the frequency nor the mean amplitude of the mEPSCs was affected by elevated levels of PRG3 distributed throughout the cell when compared to neurons with native PRG3 expression (Figure 9C).

To investigate the second part of the hypothesis that synaptic inputs might have affected the PRG3 dendrite-axonal shift, we blocked all inputs to the postsynaptic dendrites by supplementing the media with 20 μM CNQX to block AMPA/kainate receptors, 100 μM DAPV to block NMDA receptors, and 4 μM gabazine to block GABA_A_ receptors. Such treatment from DIV 4 to DIV 14 did not lead to any change in the PRG3 dendrite-axonal shift (Figure 9D). We observed a gradual localization shift of PRG3 from dendrites to axons over several days (Figure 9D). Both observations led us to conclude that the PRG3 shift is not functionally linked to the simultaneously occurring synaptic events.

## Discussion

We present novel findings regarding the role of PRG3 during neuronal development. Firstly, we provide evidence that posttranslational modification of PRG3 is essential for plasma membrane targeting and that neurite outgrowth is affected by mislocated PRG3. Secondly, we demonstrate dominant PRG3 mRNA and protein expression and localization in primary neurons that was dynamically regulated during development. Thirdly, functional analysis has revealed that PRG3 is involved in the induction of protrusions at the neurite shaft.

### Posttranslational modification of PRG3

PRG3 appeared as a glycoprotein after transfection in HEK293 cells and endogenously in mouse brain lysates. PRG3 is N-glycosylated, as predicted by *in silico* analysis and previously reported by others [20]. The N-glycosylation sequence in the second extracellular loop of PRG3 has also been identified in other LPT family members as a consensus glycosylation sequence [25,28]. In addition, our results show that N-glycosylation of PRG3 is essential for plasma membrane localization and that the latter is required for an increase of filopodia formation. However, PRG3 is also localized at intracellular membrane structures [18,20], seemingly independently of N-glycosylation.

### Endogenous PRG3 expression during development

Previous studies have shown the distribution of PRG3 transcripts in rat and mouse brains [18,19]. Furthermore, semiquantitative Northern blot analysis revealed highest mRNA expression in brain and lower levels in liver, kidney and testis [18]. These data are in line with our results, which showed approximately 10-fold higher mRNA expression levels in brain compared to liver, kidney and testis. On the protein level, PRG3 only appeared in brain samples. Therefore, we conclude that the expression level in kidney, liver and testis is below the detection level of our newly generated antibodies. In contrast, an antibody directed against a sequence located on the C-terminus of PRG3 just before the sequence used for generating anti-PRG3 296 in this study, revealed a strong PRG3 expression signal in kidney, lung, and spleen, whereas a weaker signal appeared in brain and heart tissue and hardly any signal was observed in liver [20]. However, similar to the antibodies generated by us, the latter antibody also detected PRG3 overexpression and endogenous PRG3 expression as a double band around the predicted molecular mass [20]. As we could not detect any mRNA expression in spleen tissue, the differences between these antibodies in detecting PRG3 protein expression levels cannot simply be explained by distinctions in the respective detection sensitivities of the antibodies. Further investigations are required to clarify this discrepancy.

### Functional role of PRG3

PRG3 expression is dynamically regulated after birth. We show that PRG3 mRNA levels and PRG3 protein levels decreased around ten-fold between P0 and P30. In contrast to the early embryonic expression of PRG3, PRG1, another member of the LPP-super family, is first detectable at E19 and shows a strong upregulation after birth in the hippocampus and entorhinal cortex [17]. This temporal expression pattern during brain development suggests either different or complementary functional roles of both proteins. Our data rather point to the first option. PRG1 function has been suggested as an important player in the control of hippocampal excitability [29] and it promotes outgrowth capability during regenerative sprouting [17]. However, PRG3 is expressed along all neurites in young cultured hippocampal neurons, with higher expression in the neurite shafts than in ß-actin-positive growth cones. Both a highly dynamic growth cone and a stable neurite shaft are required for neurite extensions. The neurite shaft is generated during the consolidation process, which results from the collapse of the proximal part of the growth cone and the suppression of protrusive activity along the neurite [2,8]. In addition to the localization of PRG3, our molecular data (knock-down and overexpression) lead us to propose that PRG3 has a function in the control of neurite shaft protrusion. Neurite consolidation requires constant active repression of protrusive activity [10]. We demonstrate that knock-down of PRG3 reduces the capacity of young hippocampal neurons to initiate protrusions. This, together with results of previous studies showing that PRG3 overexpression in different cells induces filopodia formation [18,20], suggests that PRG3 plays a role in the regulation of neurite shaft consolidation and could therefore control filopodia formation. Thus, PRG3 could act as a mediator for extracellular cues, which influence the initiation of neurites at the neurite shaft [30,31], therewith controlling active neurite consolidation.

Interestingly, PRG5, another member of the PRG family, induces cdc42-independent filopodia formation [32]. Whether both of these proteins act together in the process of neurite outgrowth will be clarified in the future.

In the more mature developmental stage of primary neurons, endogenous PRG3 expression switches to a mainly axon-specific expression pattern. Generally, neurites acquire either axonal or dendritic identity during development. In this process, some growth-promoting molecules like PAR1, PAR6 and phospho-GSK-3β, which had initially been expressed in all neurites, are restricted solely to one neurite, which subsequently becomes the axon [33]. Both the establishment and maintenance of neuronal polarity involve coordinated and widespread regulation of the cytoskeleton and membrane-trafficking machinery [33]. Whether PRG3 plays a role in this context remains to be examined in further studies.

Furthermore, overexpression or knock-down of PRG3 in juvenile primary hippocampal neurons induced no changes in membrane resistance or potential. This suggests that PRG3 expression and localization do not interfere with basic intrinsic neuronal properties and functions at this time point. Although their temporal and spatial similarity is striking, there appeared to be no functional coupling between dendrite-axonal shift of PRG3 and synaptic function. Our present approach targeted the capacity of neurons with pre-existing synapses to regulate the amount of new synapses or its function by dendritic PRG3 levels. At the time chosen, continuous synaptogenesis occurs with approximately 1 synapse per hour [34] with rather an upward trend [27]. However, the ability of synapses to form (initial *de novo* synaptogenesis) seems independent of PRG3, because spontaneous excitatory postsynaptic potentials were detected in cultured hippocampal neurons already at DIV 4 [35], a time point when dendrites are still packed with PRG3.

The lack of an influence on synaptic function in juvenile hippocampal neurons distinguishes PRG3 from its family member PRG1, which has been shown to influence mEPSC frequencies in the CA1 of juvenile mice [29]. Interestingly, PRG1 has also been shown to be located mainly in dendrites under non-pathological conditions. However, PRG1 protein expression was specifically upregulated in regrowing axons in the denervated outer molecular layer following lesion of the entorhinal cortex. There it attenuates phospholipid-induced axon collapse in neurons and facilitates outgrowth in the deafferented zone [17]. It remains to be elucidated whether PRG3 also plays a role in neuronal regeneration processes.

## Conclusion

In conclusion, PRG3 is highly expressed and regulated during brain development. It exhibits a mainly neuron-specific expression pattern. PRG3 is strongly expressed in the neurite shaft in young primary neurons. The knock-down of PRG3 reduces the capacity of young hippocampal neurons to initiate protrusions along the neurite shaft. Therefore, PRG3 could play a role in the regulation of active neurite consolidation. In more mature neurons, PRG3 expression is predominantly localized in the axon, and changes in PRG3 expression distribution do not affect currents.

## Abbreviations

GFAP: Glial fibrillary acidic protein; Iba1: Ionized calcium-binding adaptor molecule 1; LPA: Lysophosphatidic acid; LPPs: Lipid phosphate phosphatases; LPT: Lipid phosphatase/phosphotransferase; MAP2: Microtubule-associated protein 2; PNGase F: Peptide N-glycosidase F; PRGs: Plasticity-related genes; (LPRs: Lipid phosphatase-related protein); RT: Room temperature; tuj1: Class III β-tubulin

## Competing interests

The authors declare that they have no competing interest.

## Authors’ contributions

TV and AUB produced most of the PRG3 constructs, conducted glycosylation experiments, Western blots, immunocytochemistry co-localization study and TV produced the qRT-PCR analyses. BG, ASF and, AUB performed the morphological analysis and parts of the immunocytochemistry, AB and US designed and performed the electrophysiological experiments, and AUB designed the study and wrote the paper with contribution from all co-authors. This work was supported by DFG grant BR 2345/1-1 to AUB and the Sonnenfeld-Stiftung, which sponsored technical equipment for AUB and US. All authors read and approved the final manuscript.
